# Spatiotemporal multiple insecticide resistance in *Aedes aegypti* populations in French Guiana: need for alternative vector control

**DOI:** 10.1590/0074-02760200313

**Published:** 2021-01-29

**Authors:** Amandine Guidez, Nicolas Pocquet, Johana Restrepo, Luana Mathieu, Pascal Gaborit, Jean Issaly, Romuald Carinci, Fabrice Chandre, Yanouk Epelboin, Girod Romain, Isabelle Dusfour

**Affiliations:** 1 Institut Pasteur de la Guyane, Cayenne, French Guiana; 2 Université de Montpellier, Maladies Infectieuses et Vecteurs: Ecologie, Génétique, Evolution et Contrôle, Institut de Recherche pour le Développement, Montpellier, France; 3 Institut Pasteur de Nouvelle-Calédonie, Nouméa, Nouvelle-Calédonie; 4 Institut Pasteur de Madagascar, Antananarive, Madagascar; 5 Institut Pasteur, Paris, France

**Keywords:** Aedes aegypti, insecticide resistance, French Guiana, spatiotemporal distribution

## Abstract

**BACKGROUND:**

*Aedes aegypti* is the sole vector of urban arboviruses in French Guiana. Overtime, the species has been responsible for the transmission of viruses during yellow fever, dengue, chikungunya and Zika outbreaks. Decades of vector control have produced resistant populations to deltamethrin, the sole molecule available to control adult mosquitoes in this French Territory.

**OBJECTIVES:**

Our surveillance aimed to provide public health authorities with data on insecticide resistance in *Ae. aegypti* populations and other species of interest in French Guiana. Monitoring resistance to the insecticide used for vector control and to other molecule is a key component to develop an insecticide resistance management plan.

**METHODS:**

In 2009, we started to monitor resistance phenotypes to deltamethrin and target-site mechanisms in *Ae. aegypti* populations across the territory using the WHO impregnated paper test and allelic discrimination assay.

**FINDINGS:**

Eight years surveillance revealed well-installed resistance and the dramatic increase of alleles on the sodium voltage-gated gene, known to confer resistance to pyrethroids (PY). In addition, we observed that populations were resistant to malathion (organophosphorous, OP) and alpha-cypermethrin (PY). Some resistance was also detected to molecules from the carbamate family. Finally, those populations somehow recovered susceptibility against fenitrothion (OP). In addition, other species distributed in urban areas revealed to be also resistant to pyrethroids.

**CONCLUSION:**

The resistance level can jeopardize the efficiency of chemical adult control in absence of other alternatives and conducts to strongly rely on larval control measures to reduce mosquito burden. Vector control strategies need to evolve to maintain or regain efficacy during epidemics.

*Aedes (Stegomyia) aegypti* (Linnaeus, 1762) is a major vector of arboviruses in the tropics. In the absence of vaccines or treatment, vector control remains the main action to prevent, reduce or contain arbovirus outbreaks. Besides routine larval breeding sites reduction, insecticide applications from different molecule classes have been used against larval and adult stages of this species in succession for many years. As a consequence, resistance has developed to multiple classes of insecticide resulting in reduced efficacy at a global scale.^1^

In French Guiana, a region of the European Union in South America, *Ae. aegypti* is the only recognised vector for chikungunya, dengue, yellow fever and Zika viruses. Its distribution follows human settlements and density spreading along the coastal area, up to Maripasoula (Maroni River) and restricted to the lower Oyapock River in Saint Georges de l’Oyapock. Since the 1940s, dichlorodiphenyltrichloroethane (DDT) (organochlorine), malathion (organophosphorous) and deltamethrin (pyrethroid) were used one after another to control pest and vector mosquitoes in French Guiana.^2^ With the exception of the authorised use of malathion during the South American chikungunya outbreak in 2014, pyrethroids have been the only insecticide family authorised for adult mosquito control in France since 2011. Nowadays, deltamethrin is the cornerstone of adult control programs in French Guiana. This molecule is first used for spatial spraying applications during pest mosquito proliferation. Then, regular sprayings are applied for *Ae. aegypti* control during outbreaks. *Ae. aegypti* source reduction is also routinely performed all year long by professional teams and population is encouraged to eliminate their breeding sites in their premises. Finally, deltamethrin is indoor residual sprayed to reduce malaria transmission in hot-spots such as the Oyapock basin and inland during epidemics. Therefore, overlap between control measures against the two vectors is currently very reduced. Local vector control teams perform the pre-cited insecticide applications. In addition, pyrethroids are important compounds for long-lasting impregnated bednets distributed to prevent malaria, household sprays and pest control operated by private companies. As a result, deltamethrin resistance was recorded in *Ae. aegypti* populations in French Guiana as early as 2000.^2^ Pyrethroid pressure on mosquitoes and particularly on *Ae. aegypti* resulted in a tremendously high resistance ratio for deltamethrin.^3^ ^,^ ^4^ The historical succession of insecticide use has most likely contributed to the selection of resistant *Ae. aegypti* populations.^2^

Although mechanisms responsible for insecticide resistance are complex, resistance in mosquitoes is mainly associated with target site modification and metabolic changes. Voltage-gated sodium channel trans-membrane proteins (Na_v_) are essential for proper electrical signaling in the nervous system and other excitable cells. The toxic effect of pyrethroids modifies the function of Na_v_ by extending the activation of sodium channels and interacting with receptor sites. Mutations of pyrethroid receptor target sites in resistant mosquitoes restore this process of electrical signaling. Metabolic resistance, on the other hand, involves more subtle alterations in the expression of a complex array of enzymes and detoxification pathways. To date, studies of the resistance mechanisms in populations of *Ae. aegypti* in French Guiana have demonstrated that isoleucine amino-acid at the 1016 position (1016I) in the IIS6 region of Na_v_ and cytochrome P450 gene over-expression are strongly associated with deltamethrin resistance.^3^ ^,^ ^4^ ^,^ ^5^ ^,^ ^6^ Copy number variations (CNVs) was also associated with gene over-expression. In addition, allelic variations of enzymes demonstrate the presence of isoforms that permit higher levels of detoxification. At a larger scale, DDT and permethrin resistant *Ae. aegypti* populations exhibit a higher frequency of cysteine amino-acid at the 1534 position (1534C) of Na_v_.^7^ ^,^ ^8^ This mutation is also known to enhance deltamethrin resistance when associated to 1016I in South America.^9^ Furthermore, the two loci are known to be genetically linked and to have co-evolved.^10^ ^,^ ^11^

The study described herein presents the results of insecticides resistance monitoring in French Guiana from 2010 to 2015. Spatial and temporal distribution of alleles at the 1016 and 1534 positions of Na_v_ protein are reported in wild *Ae. aegypti* populations to assess the distribution and evolution of these mutations in French Guiana.

*Ethics statement* - Mosquito blood feeding was done on mice. Experiments were authorised by the agreement number B973-02-01 delivered by “*La préfecture de Guyane*” and renewed on June 6th, 2015. The protocol for the use of mice was approved by the ethical committee CETEA Institut Pasteur (nº 89), report number 2015-0010 issued on May 18th, 2015.

## MATERIALS AND METHODS

**Fig. 1:**
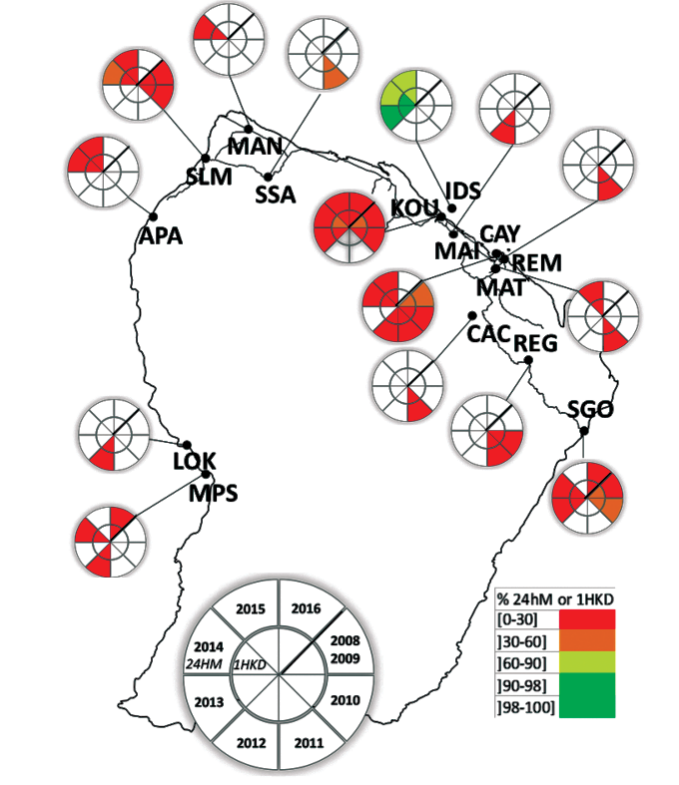
spatiotemporal distribution of knockdown effect (% 1 h KD) and mortalities (% 24 h M) in *Aedes aegypti* population against deltamethrin impregnated paper at 0.06%. Each section of pie charts represents the year, the inner circle showing the knockdown effect and the outer circle mortalities. Percent ranges are represented by a gradient of color. Mortality and KD are represented by colours along with the WHO threshold. From West to East: MPS: Maripasoula; LOK: Loka; APA: Apatou; SLM: Saint Laurent du Maroni; MAN: Mana; SSA: Saut Sabbat; KOU: Kourou; IDS: Iles du Salut; MAI: Matiti; CAY: Cayenne; REM: Rémire-Montjoly; MAT: Matoury; CAC: Cacao; REG: Régina; SGO: Saint Georges de l’Oyapock. Further details are presented in Supplementary data (Table II).

*Mosquito strains, collection and rearing conditions* - *Ae. aegypti* mosquitoes were collected in the larval stage between 2010 and 2016 in different field sites, listed by name from East to West: Saint Georges de l’Oyapock (2010, 2013, 2014, 2016), Regina (2010, 2011), Cacao (2011), Matoury (2011, 2015), Rémire-Montjoly (2011), Cayenne (2010, 2011, 2012, 2014, 2015), Matiti (2012), Kourou (2010, 2013, 2014, 2015, 2016), Iles du Salut (2013, 2014, 2015), Saut Sabbat (2011), Mana (2014), Saint-Laurent du Maroni (2010, 2014 and 2015), Apatou (2014, 2015), Loka (2012), and Maripasoula (2012, 2014, 2016) (Fig. 1). Saint Georges de l’Oyapock, Cayenne, Kourou and Saint Laurent du Maroni are considered towns while others are villages. Older data collected in 2008 and 2009 and published in Dusfour et al.^3^ were added to the analyses, especially Fig. 1. F1 generation were exposed to the insecticide.

Populations of *Culex quinquefasciatus* were collected in the larval stage in Kourou, Saint Georges de l’Oyapock and Rémire-Montjoly in 2016. Adults of the F0 generation were reared and allowed to mate. Females were provided a blood meal from mice to collect eggs of the F1 generation. Adults of the F1 generation were then obtained by rearing immature stages concurrently under insectary conditions (28 ± 1ºC, 70 ± 10% RH, 12:12h photoperiod) in French Guiana.

*Aedes taeniorhynchus* females were collected in Cayenne, fed on mice and allowed to lay eggs. Following generations were allowed to reproduce as previously mentioned. The F2 generation of that species was used for testing.

*Assessment of insecticide resistance* - Insecticide resistance bioassays were carried out for each population through tarsal contact tests using filter papers impregnated with technical grade compounds (Sigma-Aldrich, St Louis, MO, USA): deltamethrin (CAS # 52918-63-5) (Sigma-Aldrich, St Louis, MO, USA) at the diagnostic dose of 0.06% for *Aedes* sp. and 0.025% for *Culex* sp., alpha-cypermethrin (#45806, Sigma-Aldrich) at the diagnostic dose of 0.05%, malathion (#45614, Sigma-Aldrich) at the dose of 0.8 and 5%, fenitrothion (#45487, Sigma-Aldrich) at the diagnostic dose of 0.5%, propoxur (#45644, Sigma-Aldrich) at the diagnostic dose of 0.1% and bendiocarb (#45336, Sigma-Aldrich) at the diagnostic dose of 0.1%. Filter papers were impregnated using acetone solutions of insecticide and silicone oil or olive oil^12^ by dropping 2 mL of the solution onto each paper (12 x 15 cm). Doses are expressed in weight/volume (w/v) percentage of the active ingredient in silicone oil. The papers were dried for 24 h before the test. For each population, four batches of 25 non-blood fed, 2-5 day old females were introduced into holding tubes for one hour then transferred into the exposure tube and placed vertically for 1 h.^13^ Knocked-down (*kd*) and dead mosquitoes were recorded after this time (1 h KD) before being transferred back to the holding tubes. A mosquito was recorded as knocked-down if it was lying on its back or side and was unable to instigate flight after a gentle tap. Mortality was recorded 24 h after exposure (24 h M). Controls, made of only acetone and silicone oil delivered to the filter paper, were performed as mentioned above with a total of two replicates of 25 non-blood fed 2-5 day old females. Females from PAEA and New Orleans reference strains were raised under identical insectary conditions and used as susceptible controls. All replicates were conducted at 27 ± 2ºC and 60 ± 10% RH. A ten percent sugar solution was provided to the females during the 24-h observation period in holding tubes.

WHO protocols for insecticide resistance monitoring provide a diagnostic threshold of resistance.^13^ Any population is considered susceptible when the percentage of mortality is above 98% after exposure to an impregnated paper at the diagnostic dose. Mortality below 98% is suggestive of resistance; below 90%, resistance is confirmed; between 90% and 97% tests must be triplicated and individuals tested for molecular resistance markers. When several tests were performed during one-year results of mortality or *kd* were aggregated by means.

*Genotyping V1016I and F1534C loci* - Each adult mosquito was grinded individually by hand with a sterile grinder. DNA was extracted using either the Purelink Genomic DNA extraction kit (Invitrogen, Carlsbad, CA, USA) or DNeasy Blood & Tissue Kit (Qiagen, Hilden, Germany) according to the manufacturer’s instructions or Collins et al.^14^ The DNA isolated was quantified using a spectrophotometer (NanoDrop 2000c, Thermo Scientific, Waltham, MA, USA) and a 10-fold dilution was used for polymerase chain reaction (PCR). DNA was then stored at -20ºC.

Mosquitoes from each locality were genotyped at both 1016 (Val/Ile) and 1534 (Cys/Phe) positions from genomic DNA by real time PCR allelic discrimination assay (Applied Biosystems, CA, USA). Two primers and two minor groove binding probes were designed for V1016I detection as previously described.^6^ A second amino acid substitution, F1534C, was targeted on the IIIS6 region of the Na_v_ gene according to Yanola et al.^8^ Primers and probe sequences are listed in Supplementary data (Table I). Each allelic discrimination assay was carried out with 12.5 µL of 2X TaqMan^*®*^ Universal Master Mix II (Life Technologies, Gaithersburg, MD, USA), 1.44 µM of primers, 0.4 µM of each of the specific probes and 3 µL of genomic DNA (20 ng), in a total volume of 25 µL. Thermocycling amplification conditions were 10 min at 95***º*** C followed by 45 cycles of 95***º*** C for 15 s and 60***º*** C for 1 min. The amplification reactions were performed in a StepOnePlus Real-Time PCR System (Life Technologies, Gaithersburg, MD, USA) and analyses were done by the StepOne Software version 2.1.

The cor.test function in R 3.2.2 (R Development Core Team, Vienna, Austria) was used to find the (*r*) correlation between percent mortalities and frequency of alleles or genotypes. Dilocus haplotypes 1016/1534 appear in four possible forms: V1016/F1534 (VF), V1016/C1534 (VC), I1016/F1534 (IF), I1016/C1534 (IC). In double heterozygotes, the bi-allelic combination is unknown. Therefore, the composite disequilibrium frequencies and tested linkage disequilibrium were determined as described previously.^11^ In addition, variance analysis was performed to evaluate the effect of each genotype on the resistance phenotypes using aov function in R 3.2.2 (R Development Core Team, Vienna, Austria). Data from Dusfour et al.^3^ were used as a baseline for spatio-temporal analyses.

## RESULTS

*Selection of deltamethrin resistance in French Guiana* - Since 2009, resistance monitoring has been conducted for *Ae. aegypti* populations across their distribution in French Guiana in a total of 15 localities (Fig. 1). Nine of the populations have been monitored two or more times over that period (Fig. 1). The more populated towns [Saint Laurent du Maroni (SLM), Kourou (KOU), Cayenne (CAY), Saint Georges de l’Oyapock (SGO)] were sampled several times with fewer collections in small and remote villages. Prior monitoring showed that deltamethrin resistance was already established in 2008-2009 in the main coastal towns.^3^ Consecutive follow-up in hamlets in littoral zones [Matiti (MAI), Saut Sabbat (SSA)] or in remote inland villages [Loka (LOK), Maripasoula (MPS)] revealed that high resistance was widespread across the territory with monitoring results showing mortality below 40% mainland. The most noticeable result is the susceptibility observed from a mosquito population on Iles du Salut (IDS) in 2013 with a subsequent loss of susceptibility the following years [Fig. 1, Supplementary data (Table II)]. In the main towns, mortality and knockdown effect dropped below 5% for CAY, KOU and SLM.

**Fig. 2:**
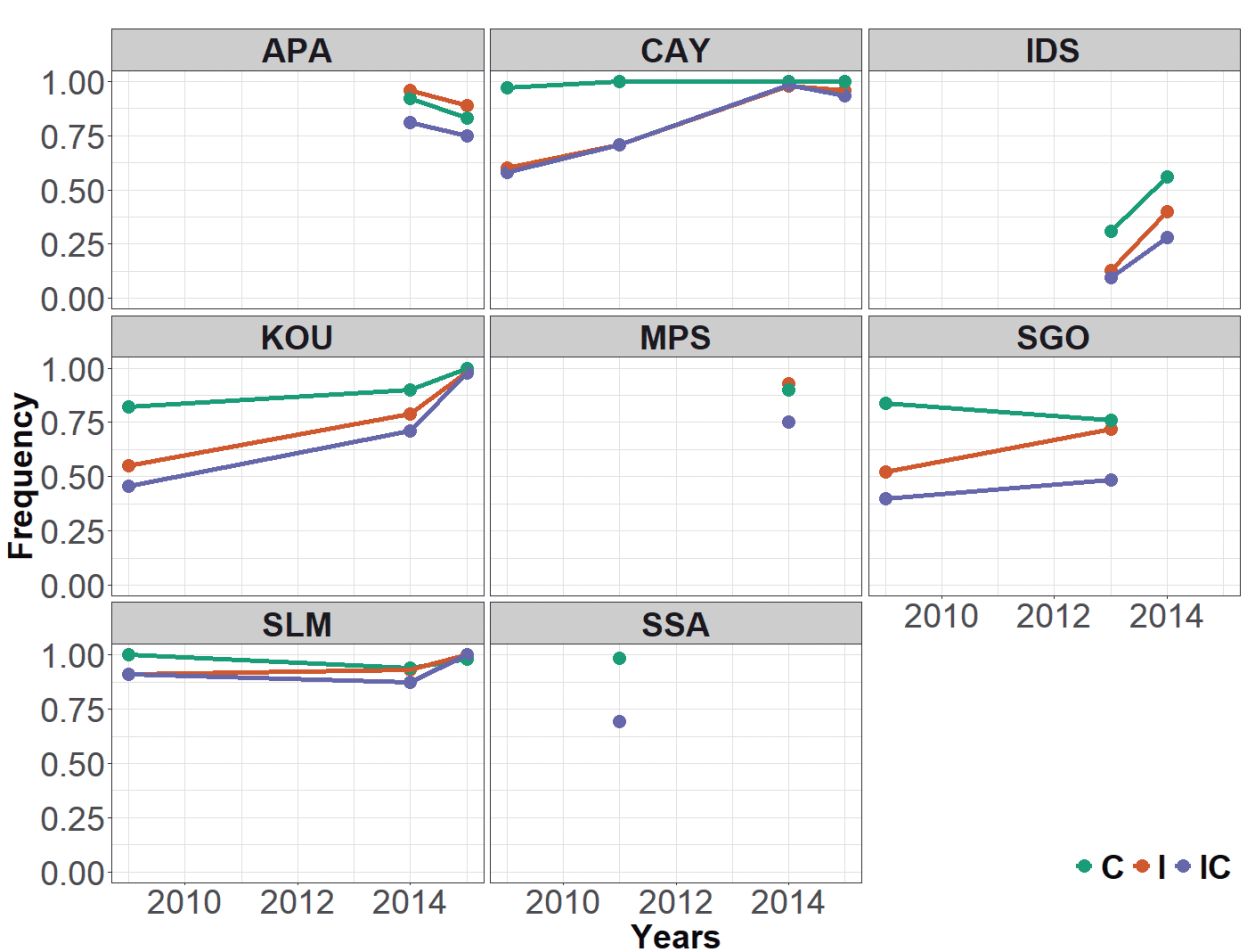
frequencies of resistant alleles at 1534 (C) and 1016 (I) loci separately and combined (IC), per year and per site. MPS: Maripasoula; APA: Apatou; SLM: Saint Laurent du Maroni; SSA: Saut Sabbat; KOU: Kourou; IDS: Iles du Salut; CAY: Cayenne; SGO: Saint Georges de l’Oyapock.

*Resistant alleles and double resistant genotypes are reaching fixation* - A total of 621 and 567 samples were genotyped for loci 1534 and 1016 respectively, and 550 with the double genotyping. Overall, observed frequencies of the resistant 1016I varied from 0.52 to 1.00 while those of 1534C allele ranged from 0.76 to 1.00 in continental populations [Supplementary data (Table III), Fig. 2]. As expected, 1016I frequencies were lower on IDS at 0.13 and 0.40 in 2013 and 2014 respectively. Frequencies of 1534C in IDS were 0.31 and 0.56 in 2013 and 2014 respectively.

In 2009, 1534C frequencies were already high, above 0.80, in the main town and have almost reached fixation in SLM, KOU and CAY as of the last recorded assay (Fig. 2). 1016I frequencies were lower than 1534C for those populations and tended to increase over time. SLM populations exhibited high frequencies throughout all periodic assays (> 0.80). In examining the whole dataset, independent frequencies of those alleles correlated negatively with percent mortality and knocked-down mosquitoes in resistance assays (Table I). As expected, significant linkage disequilibrium was observed between the two loci (p < 0.001). Therefore, both loci were combined for further analyses. Biallelic resistant haplotype increased in the same pattern as independent alleles and is also significantly correlated with the phenotypes (Table I).

**TABLE I:** Results of the Pearson correlation test between resistance alleles and knockdown /mortality effects

Alleles/genotypes	1 h KD			24 h M		
	*r*	p-value	CI 95%	*r*	p-value	CI 95%
1534C	-0.76	0.0001	-0.90; -0.47	-0.76	0.0002	-0.90; -0.46
1016I	-0.89	< 0.0001	-0.95;-0.72	-0.80	< 0.0001	-0.92; -0.53
1016I/1534C	-0.80	< 0.0001	-0.92;-0.54	-0.72	0.0007	-0.88;-0.38

CI 95%: confidence interval 95%.

**Fig. 3:**
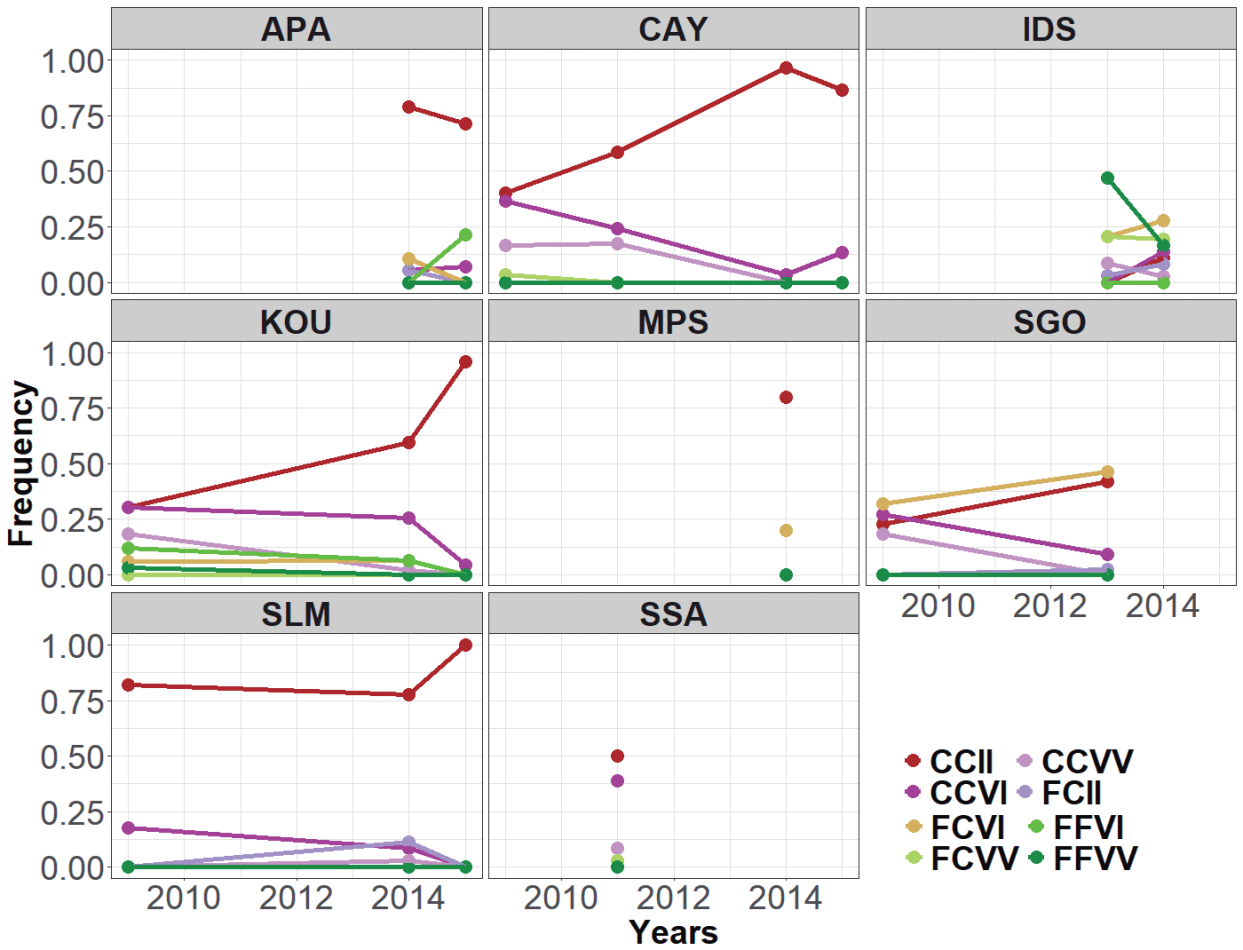
frequencies of combined genotypes at 1534 and 1016 loci, per year and per site. MPS: Maripasoula; APA: Apatou; SLM: Saint Laurent du Maroni; SSA: Saut Sabbat; KOU: Kourou; IDS: Iles du Salut; CAY: Cayenne; SGO: Saint Georges de l’Oyapock. CCII: homozygous mutant CC1534/homozygous mutant II1016; CCVV: homozygous mutant CC1534/wild-type VV1016; FCVI: heterozygous FC1534/heterozygous VI1016; FFVI: wild-type FF1534/heterozygous VI1016; CCVI: homozygous mutant CC1534/heterozygous VI1016; FCII: heterozygous FC1534/homozygous mutant II1016; FCVV: heterozygous FC1534/wild-type VV1016; FFVV: wild-type FF1534/wild-type VV1016.

Double mutant genotype (CCII) is known to confer a higher selective advantage under insecticide pressure [Supplementary data (Table III), Fig. 3]. This highly resistant genotype was maintained or increased over time the last decade, in accordance with deltamethrin phenotype of mortality (*r* = -0.73, p-value < 0.0001) and knock-down (*r* = -0.82, p-value < 0.0001) [Supplementary data (Table III)]. IDS and KOU are the only populations exhibiting double homozygote susceptible but whose frequency decreased.

Variance analysis statistically confirmed the dominant effect of CCII genotype but also showed that CCVI, FCVV and FFVV have an effect on *kd* phenotype of the populations while CCII, CCVI, FCII and FCVV contribute to the mortality phenotypes. Overall genotypes contribute higher to *kd* phenotypes than to mortality [Supplementary data (Table IV)].

*Ae. aegypti phenotypes for other insecticides* - Over the years, sporadic checks of resistance to other molecules from the main insecticide families have been performed (Table II). 24-h mortalities against alpha-cypermethrin were even lower than the score for deltamethrin mortality. Bendiocarb (carbamate) demonstrated a loss of susceptibility while propoxur from the same insecticide family demonstrated resistance according to WHO recommended thresholds. *Ae. aegypti* populations seemed to regain susceptibility against fenitrothion in CAY and SGO where they were assayed at several time points. Susceptibility for that molecule remained in MPS between 2012 and 2016 while resistance has not changed in KOU.

**TABLE II:** Mean percentages of mortality and standard deviation at 24 h for impregnated paper tests per insecticide, *Aedes aegypti* population and year. The total number of exposed mosquitoes are indicated (n). Data from the years 2008 and 2009 are from Dusfour et al.^3^

Molecule	Dose	Population	Year	n	Mean 24 h M (%)	SD (%)
Alphacypermethrin	0.05%	CAY	2016	100	5.00	5.03
		KOU	2016	100	3.00	3.82
		MPS	2016	100	0.00	0.00
		SGO	2016	100	0.00	0.00
Bendiocarb	0.1%	MPS	2016	100	91.00	5.03
		SGO	2016	100	96.00	3.26
Propoxur	0.1%	CAY	2011	102	13.69	1.95
		MPS	2016	100	76.00	3.26
		SGO	2016	100	76.00	3.26
Fenitrothion	0.5%	CAY	2009	198	43.85	12.44
			2011	101	74.41	13.53
			2016	100	94.00	4.00
		KOU	2009	212	47.65	15.32
			2016	100	45.00	1.99
		MPS	2012	98	100.00	0.00
			2016	100	100.00	0.00
		SGO	2008	321	29.30	18.54
			2016	100	96.00	4.61
		SLM	2009	199	85.40	7.98
Malathion	0.8%	CAY	2014	196	31.88	26.47
		KOU	2014	192	9.07	7.81
			2015	95	4.16	3.40
		MAT	2014	124	20.86	11.7
			2015	97	13.47	8.70
		SGO	2008	99	12.12	3.27
			2014	191	8.89	5.52
			2015	100	4.92	4.81
		SLM	2015	98	10.25	7.80
	5%	CAY	2014	195	99.47	1.47
		KOU	2014	199	98.5	2.07
			2015	94	94.65	5.20
		MAT	2015	95	100.00	0.00
		SGO	2014	100	100.00	0.00
			2015	102	87.66	14.86
		SLM	2015	98	100.00	0.00

CAY: Cayenne; KOU: Kourou; MPS: Maripasoula; SGO: Saint Georges de l’Oyapock; SLM: Saint Laurent du Maroni; MAT: Matoury. SD: standard deviation.

Inconsistencies occurred in the follow-up on malathion resistance. Indeed, the diagnostic dose of 0.8% did not succeed in killing 100% of the reference strains (data not shown). The diagnostic dose was then complemented by a 5% dose, formerly used for that species and currently applied for other mosquito species*.* Then malathion resistance was confirmed to be recorded in *Ae. aegypti* populations from French Guiana.

*Resistance in other urban mosquitoes* - In a context of emergent arboviruses, the resistance of two other urban mosquito species was tested against deltamethrin in 2016 and 2017 (Table III). In absence of any reference strain for *Ae. taeniorhynchus,* the diagnostic dose established for *Ae. aegypti* was used. The test detected resistance in the population from Cayenne. Populations of *Cx. quinquefasciatus* from Kourou, Rémire-Montjoly and Saint Georges de l’Oyapock also presented high resistance to deltamethrin.

**TABLE III:** Mean percentage of *kd* effect and mortality with standard deviation for impregnated paper tests with deltamethrin 0.06% (*Aedes*) and 0.025% (*Culex*) per population. The total number of exposed mosquitoes are indicated (n)

Species	Locality	n	% 1 h KD	SD (%)	% 24 h M	SD (%)
*Aedes taeniorhynchus*	CAY	101	46.93	20.02	17.14	11.10
*Culex quinquefasciatus*	KOU	100	1.00	2.00	1.00	2.00
	REM	97	14.43	3.06	5.17	2.51
	SGO	100	3.00	3.80	9.00	3.82

CAY: Cayenne; KOU: Kourou; REM: Rémire-Montjoly; SGO: Saint Georges de l’Oyapock. SD: standard deviation.

## DISCUSSION

Monitoring resistance to insecticides is a key component in sustainable vector control and the development of a resistance management program.^15^ French Guiana is an overseas territory of France, located in South America but subject to European and French biocide regulations. In the absence of a vaccine or treatment for arboviruses, *Ae. aegypti* control had been implemented by reducing or treating breeding sites all year, and by insecticide spraying during dengue and other arbovirus transmission. After the reintroduction of DDT-resistant *Ae. aegypti* population in the 1950s, malathion, an organophosphorous, was used for outdoor spatial spraying for decades. Later, deltamethrin, a molecule from the pyrethroid family, was also used for indoor spatial sprays around dengue cases. Temephos (organophosphorous) was used as larvicide succeeded by *Bacillus thuringiensis* var *israelensis* in 2000 along with larval source reduction measures. In 2011, deltamethrin became the only authorised and available insecticide for indoor and outdoor adult mosquito control. In 2014, a chikungunya outbreak hit the territory and *Aedes* control was intensified with weekly ground-spatial sprays of deltamethrin but also with malathion, exceptionally implemented for six months during this epidemic.^2^ The Zika outbreak followed in 2016, during which only deltamethrin was used for spraying. These periodic outbreaks with intensified insecticide use are the times when heavy selective pressure occurs, but other indirect sources of pyrethroid exposure exist. In fact, control of malaria or pest mosquitoes is conducted with recurring indoor residual spraying, distribution of pyrethroid-impregnated bed nets and spatial spraying which also exert non-negligible insecticide pressures on the mosquito populations. In addition, the use of household sprays is frequent and suspected to play a role in resistance selection by pyrethroids in urban mosquito populations.^16^ No robust data exists to support the foregoing hypothesis in French Guiana before 2009. The selection caused by insecticides in urban environments needs to be studied as a whole to explain resistance patterns of current and potential vectors at local and global scales.^17^ ^,^ ^18^

Even though it is difficult to decipher the dominant factor inducing resistance to deltamethrin, high levels were already recorded in *Ae. aegypti* populations at the early stage of its use for spatial spraying in 2011.^3^ At that time, both resistance to deltamethrin and DDT were documented in French Guiana.^2^ Cross resistance is often observed between pyrethroids and DDT^19^ ^,^ ^20^ due to the same target sites of these molecules, even if recent meta-analysis data in the geographical region does not concur.^21^ The continuous pressure by either one or both molecules before 2011 probably maintained the level of resistance to deltamethrin and other pyrethroids in *Ae. aegypti* populations and other urban mosquitoes in areas where these compounds are used for malaria control by indoor residual spraying (i.e. Saint Georges de l’Oyapock and Saint Laurent du Maroni). However, low gene flow and local selection of resistance should have shaped different resistance patterns amongst populations.^22^ ^,^ ^23^ In contrast, malathion and temephos were the mainstay of vector and pest mosquito control in the central littoral strip, where malaria is a rare event for the last 40 years.

However, spatio-temporal monitoring of deltamethrin resistance demonstrates the widespread distribution of the phenotypes and its important increase globally, reaching mortality lower than 5% in 2015-2016 with the exception of the population on Iles du Salut, whose resistance development appears later than on the mainland. This archipelago is a unique location where vector or pest control is rarely performed and is connected with Kourou on the continent by boat and helicopter only.

Our monitoring also showed a great variability amongst results probably a consequence of the intensification of domestic, and uncontrolled, use of pyrethroids during periods of high infestation as already been evidenced.^24^

The investigation of molecular markers related to resistance at positions 1016 and 1534 of the Na_v_ gene demonstrated a strong correlation between resistant alleles alone or in combination with percent of knocked-down mosquitoes and mortality. These results are in agreement with previous data observed in French Guiana.^4^ ^,^ ^5^ ^,^ ^6^ The resistant 1534C was already at high frequencies, almost to fixation, in 2009 compared to 1016I, whose frequencies were below 50% across the studied populations except for Saint Laurent du Maroni, where frequency was already above 90%. This observation has been made in other countries where 1534C is suspected to have appeared before 1016I.^11^ The appearance of 1534C could be associated with DDT resistance to which that mutation confers a selective advantage greater than for pyrethroids.^11^ The rapid expansion of 1016I is then related to the co-evolution of the two loci, the presence of 1534C and in particular the genotype CCIV in initial populations had most likely been crucial as the double homozygote resistant gives a strong advantage under deltamethrin pressure.^9^

In addition, resistant alleles are partially recessive and the presence of heterozygous genotypes do not confer full resistance.^10^ ^,^ ^25^ Four different patterns can be observed in genotypes. First, the population from Saint Laurent du Maroni was already close to fixation in 2009. Then, genotype evolution in Saint Georges de l’Oyapock was composed of more frequent double heterozygotes or 1534 heterozygotes than in other locations. In addition, Cayenne and Kourou, in the centre of the littoral strip, have similar patterns of evolution with the rapid expansion of double resistant homozygotes while C1534 was already in place. Finally, Iles du Salut was drastically different and the double homozygotes susceptible recorded in 2013 decreased to favor heterozygote or homozygote resistant individuals. The primary cause is most likely genetic structure among populations of French Guiana, inducing a local selection of resistance and therefore the development of different patterns.^22^ ^,^ ^23^ These selective pressures with either pyrethroids or DDT are not easy to unravel. However, the fact that Saint Laurent du Maroni and Saint Georges de l’Oyapock have been subjected to both malaria vector control and *Aedes* control must be noted. In opposite, Kourou and Cayenne were poorly impacted by malaria control due to low transmission. A few malaria hot-spots were sporadically treated in both towns. Iles du Salut is a unique setting without spraying except occasional pest treatment. Another study shows local structure of resistance in *Ae. aegypti*, reinforcing the difficulty to monitor and predict their development.^26^ Even with the high importance of *kdr* mutation in deltamethrin phenotype resistance, previous investigations have demonstrated the implication of detoxification enzymes in French Guiana populations and particularly genes from the CYP450 gene.^4^ ^,^ ^5^ ^,^ ^6^

Recent data on those particular populations demonstrate that other CYP450 genes are also associated with fenitrothion (organophosphorous) and bendiocarb (carbamates)^27^ resistance in the absence of mutations on the acetylcholinesterase gene,^4^ the target of organophosphorous and carbamates. This result concurs with the low cross-resistance between deltamethrin and bendiocarb and propoxur and the susceptibility recovery for fenitrothion we observed. Cross-resistance was also observed between pyrethroid, malathion and temephos whose resistance selection by one another has been demonstrated.^28^ ^,^ ^29^ ^,^ ^30^ The persistence of malathion resistance in the presence of pyrethroid pressure and vice-versa before 2009 in French Guiana support these observations.

Beyond the *Ae. aegypti* populations resistance patterns, resistance to pyrethroids occurs for *Cx. quinquefasciatus*, and *Ae. taeniorhynchus*, pest mosquitoes in French Guiana but competent to transmit other arboviruses.

Effective urban mosquito control with pyrethroids has come to a dead-end in French Guiana due to high levels of resistance across the country. Alternative methods of vector and pest control should be investigated to eventually regain susceptibility, reinforce larval control and support a resistance management plan.
