# Supplementary material for: Spatiotemporal multiple insecticide resistance in Aedes aegypti populations in French Guiana: need for alternative vector control
Source: Mem Inst Oswaldo Cruz. 2021 Jan 29;115:e200313. doi: 10.1590/0074-02760200313 (PMC7849183; doi:10.1590/0074-02760200313)
Supplement: Supplementary file 1 [file 1678-8060-mioc-115-e200313-s.pdf]

TABLE I

List of primers and probes for *kdr* Allelic Discriminant Assays

Loci	Primer name	Primer sequences (5'> 3')	Primer size (bp)	Probe name	Probe sequences (5'> 3')	Probe size (bp)
IIS6-1016	1016F	GCT-AAC-CGA-CAA-ATT-GTT-TCC-C	22	V1016	(VIC)-CAC-AGG-TAC-TTA-ACC-TTT-T-(MGBNFQ)	19
	1016R	CAG-CGA-GGA-TGA-ACC-GAA-AT	20	I1016	(FAM)-CAC-AGA-TAC-TTA-ACC-TTT-TC-(MGBNFQ)	20
IIIS6-1534	1534F	GAT-GAT-GAC-ACC-GAT-GAA-CAG-ATC	24	F1534	(VIC)-AAC-GAC-CCG-AAG-ATG-A-(MGBNFQ)	16
	1534R	CGA-GAC-CAA-CAT-CTA-GTA-CCT	21	C1534	(FAM)-ACG-ACC-CGC-AGA-TGA-(MGBNFQ)	15

TABLE II

Knocked-down (1 h KD) and dead mosquitoes (24 h M) exposed to deltamethrin 0.06% in the the impregnated paper test represented in mean percentage (%) per locality, month and year

					1 h KD			24 h M		
Year	Population	Month	n	N	%	SD	SEM	%	SD	SEM
2010	Cayenne	Sept	100	4	24.9	0.076	0.038	23.9	0.122	0.061
	Kourou	Oct	99	4	15.1	0.037	0.019	17.1	0.058	0.029
	Regina	April	101	4	6.8	0.066	0.033	23.6	0.101	0.050
	Saint Georges de L'Oyapock	Oct	100	4	30.1	0.086	0.043	40.1	0.080	0.040
	Saint Laurent du Maroni	Oct	97	4	20.6	0.057	0.028	25.8	0.091	0.046
2011	Cacao	May	80	4	8.8	0.075	0.038	10.0	0.058	0.029
	Cayenne	Feb	150	14	22.7	0.133	0.054	39.3	0.185	0.075
		May	99	4	29.2	0.130	0.065	22.3	0.136	0.068
		Sept	93	8	9.6	0.040	0.020	14.0	0.055	0.028
	Matoury	Dec	98	4	10.2	0.089	0.044	20.5	0.082	0.041
	Regina	March	97	6	2.3	0.069	0.035	1.9	0.054	0.027
		Nov	97	4	0.0	0.051	0.026	1.0	0.039	0.019
	Remire-Montjoly	June	94	4	22.6	0.052	0.026	22.4	0.123	0.062
	Saut Sabbat	Feb	135	4	43.2	0.045	0.023	36.8	0.033	0.017
2012	Cayenne	June	100	4	2.0	0.000	0.000	2.0	0.021	0.010
	Loka	June	100	8	13.0	0.111	0.055	14.0	0.063	0.031
	Maripasoula	June	98	4	9.1	0.108	0.044	6.1	0.192	0.079
	Matiti	Aug	99	8	10.1	0.030	0.011	7.1	0.045	0.016
2013	Iles du Salut	May	200	4	98.0	0.090	0.045	92.5	0.076	0.038
	Kourou	Nov	99	11	11.2	0.105	0.053	9.1	0.161	0.081
	Saint Georges de L'Oyapock	March	100	13	21.0	0.024	0.012	21.0	0.041	0.021
		Sept	98	8	2.1	0.068	0.034	3.2	0.171	0.085
2014	Apatou	Sept	99	4	7.0	0.120	0.060	15.2	0.245	0.122
	Cayenne	Dec	99	3	14.6	0.120	0.045	22.6	0.101	0.038
		Sept	259	8	31.6	0.306	0.176	25.3	0.247	0.142
	Iles du Salut	Sept	232	4	94.0	0.038	0.012	82.7	0.108	0.034
	Kourou	Sept	195	4	33.6	0.261	0.092	17.8	0.104	0.037
	Mana	Sept	92	4	8.4	0.058	0.029	12.0	0.075	0.037
	Maripasoula	Sept	77	13	6.6	0.084	0.048	14.4	0.085	0.049
	Saint Georges de L'Oyapock	Dec	99	4	8.1	0.076	0.038	12.0	0.118	0.059
		Sept	100	4	27.2	0.078	0.039	10.1	0.074	0.037
	Saint Laurent du Maroni	Sept	97	4	7.2	0.085	0.042	42.3	0.054	0.027
2015	Apatou	June	95	4	3.1	0.021	0.010	10.7	0.025	0.013
	Cayenne	Oct	105	4	2.8	0.019	0.010	4.8	0.019	0.010
	Iles du Salut	Jan	312	4	85.6	0.127	0.035	64.1	0.171	0.047
	Kourou	June	98	4	0.0	0.000	0.000	1.0	0.038	0.019
	Matoury	June	97	4	9.5	0.115	0.058	17.0	0.286	0.143
	Saint Laurent du Maroni	June	95	4	0.0	0.000	0.000	3.1	0.034	0.017
2016	Kourou	July	100	4	7.0	0.020	0.010	3.0	0.038	0.019
	Maripasoula	July	100	4	7.0	0.038	0.019	0.0	0.000	0.000
	Saint Georges de L'Oyapock	June	99	4	29.2	0.126	0.063	11.2	0.022	0.011

SD: standard deviation; SEM: standard error to the mean. The number of mosquitoes tested is indicated (n) and the number of replicates by N.

TABLE III

Genotypes frequencies and number of mosquito tested (n) at both loci per locality and year

			Genotypes							
Localities	Years	n	CCII	CCVI	CCVV	FCII	FCVI	FCVV	FFVI	FFVV
CAY	2009	30	0.40	0.37	0.17	0.00	0.03	0.03	0.00	0.00
	2011	29	0.59	0.24	0.17	0.00	0.00	0.00	0.00	0.00
	2014	30	0.97	0.03	0.00	0.00	0.00	0.00	0.00	0.00
	2015	15	0.87	0.13	0.00	0.00	0.00	0.00	0.00	0.00
IDS	2013	34	0.00	0.00	0.09	0.03	0.21	0.21	0.00	0.47
	2014	36	0.11	0.14	0.03	0.08	0.28	0.19	0.00	0.17
KOU	2009	33	0.30	0.30	0.18	0.00	0.06	0.00	0.12	0.03
	2014	47	0.60	0.26	0.02	0.00	0.06	0.00	0.06	0.00
	2015	24	0.96	0.04	0.00	0.00	0.00	0.00	0.00	0.00
SGO	2009	44	0.23	0.27	0.18	0.00	0.32	0.00	0.00	0.00
	2013	43	0.42	0.09	0.00	0.02	0.47	0.00	0.00	0.00
SLM	2009	28	0.82	0.18	0.00	0.00	0.00	0.00	0.00	0.00
	2014	36	0.78	0.08	0.03	0.11	0.00	0.00	0.00	0.00
	2015	17	1.00	0.00	0.00	0.00	0.00	0.00	0.00	0.00
SSA	2011	36	0.50	0.39	0.08	0.00	0.00	0.03	0.00	0.00
APA	2014	19	0.79	0.05	0.00	0.05	0.11	0.00	0.00	0.00
	2015	14	0.71	0.07	0.00	0.00	0.00	0.00	0.21	0.00
MPS	2014	35	0.80	0.00	0.00	0.00	0.20	0.00	0.00	0.00

CAY: Cayenne; IDS: Iles du Salut; KOU: Kourou; SGO: Saint Georges de l'Oyapock; SLM: Saint Laurent du Maroni; SSA: Saut Sabbat; APA: Apatou; MPS: Maripasoula.

TABLE IV

Output from variance analyses in R

```
> fit4 <-aov(KD~CCII+CCVI+CCVV+FCII+FCVI+FCVV+FFVI+FFVV. data=spe)
> summary(fit4)
            Df Sum Sq Mean Sq F value   Pr(>F)
CCII         1 0.8021  0.8021  95.858 4.27e-06 ***
CCVI         1 0.1978  0.1978  23.633 0.000894 ***
CCVV         1 0.0009  0.0009   0.107 0.751595
FCII         1 0.0158  0.0158   1.889 0.202592
FCVI         1 0.0055  0.0055   0.659 0.437697
FCVV         1 0.2267  0.2267  27.086 0.000561 ***
FFVI         1 0.0020  0.0020   0.240 0.635638
FFVV         1 0.0412  0.0412   4.923 0.053676 .
Residuals    9 0.0753  0.0084
---
Signif. codes:  0 '***' 0.001 '**' 0.01 '*' 0.05 '.' 0.1 ' ' 1


> fit4b <-aov(KD~CCII+CCVI+FCVV+FFVV. data=spe)
> summary(fit4b)
            Df Sum Sq Mean Sq F value   Pr(>F)
CCII         1 0.8021  0.8021 103.844 1.44e-07 ***
CCVI         1 0.1978  0.1978  25.602 0.000219 ***
FCVV         1 0.2321  0.2321  30.048 0.000105 ***
FFVV         1 0.0349  0.0349   4.514 0.053368 .
Residuals   13 0.1004  0.0077
---
Signif. codes:  0 '***' 0.001 '**' 0.01 '*' 0.05 '.' 0.1 ' ' 1


> fit5 <-aov(M~CCII+CCVI+CCVV+FCII+FCVI+FCVV+FFVI+FFVV. data=spe)
> summary(fit5)
            Df Sum Sq Mean Sq F value   Pr(>F)
CCII         1 0.5794  0.5794  68.613 1.67e-05 ***
CCVI         1 0.0549  0.0549   6.507  0.03115 *
CCVV         1 0.0029  0.0029   0.339  0.57496
FCII         1 0.1604  0.1604  19.002  0.00183 **
FCVI         1 0.0081  0.0081   0.964  0.35175
FCVV         1 0.1479  0.1479  17.512  0.00236 **
FFVI         1 0.0005  0.0005   0.060  0.81138
FFVV         1 0.0151  0.0151   1.790  0.21378
Residuals    9 0.0760  0.0084
---
Signif. codes:  0 '***' 0.001 '**' 0.01 '*' 0.05 '.' 0.1 ' ' 1

> fit5b <-aov(M~CCII+CCVI+FCII+FCVV. data=spe)
> summary(fit5b)
            Df Sum Sq Mean Sq F value   Pr(>F)
CCII         1 0.5794  0.5794  59.474 3.33e-06 ***
CCVI         1 0.0549  0.0549   5.641  0.03362 *
FCII         1 0.1427  0.1427  14.649  0.00210 **
FCVV         1 0.1416  0.1416  14.536  0.00215 **
Residuals   13 0.1266  0.0097
---
Signif. codes:  0 '***' 0.001 '**' 0.01 '*' 0.05 '.' 0.1 ' ' 1
>
```
